# Exploring the Mediating Role of Situation Awareness and Crisis Emotions Between Social Media Use and COVID-19 Protective Behaviors: Cross-Sectional Study

**DOI:** 10.3389/fpubh.2022.793033

**Published:** 2022-04-28

**Authors:** Yulei Feng, Qingyan Tong

**Affiliations:** School of Media and Communication, Shanghai Jiao Tong University, Shanghai, China

**Keywords:** COVID-19, social media, crisis emotions, preventive action, cross-sectional study

## Abstract

**Background:**

In an outbreak of an infectious disease especially, online media would usually be an important channel for people to get first-hand knowledge and evaluate risks of the specific emergency. Although there has been increasing attention to the effect of social media use during epidemics and outbreaks, relatively little is known about the underlying mechanism by which social media plays a role in people's cognitive, affective and preventive responses.

**Objective:**

With an objective to advance current knowledge surrounding social media effects on people's cognition, affection and health protective behaviors during epidemics, we aim to examine the associations between social media exposure to COVID-19 risks related information and preventive behavior of the public, and also the role that situation awareness and crisis emotions including anxiety and fear played.

**Methods:**

An online cross-sectional survey was conducted in China a total of 632 participants were recruited. Measures included exposure to COVID-19 information through social media, situation awareness, anxiety, fear and protective behaviors that participants have taken. We have performed the descriptive statistical analysis, correlation and mediation analysis to test the research hypotheses.

**Results:**

Findings indicated that situation awareness was positively associated with social media use (B = 0.54, *p* < 0.001), anxiety (B = 0.95, *p* < 0.001) and fear (B = 0.87, *p* < 0.001), and preventive behavior (B = 0.68, *p* < 0.001). Social media use would also have an indirect effect on anxiety (indirect effect = 0.40; 95% CI = [0.34, 0.46]) and fear (indirect effect = 0.35; 95% CI = [0.29, 0.42]) through situation awareness. The serial mediation effect of situation awareness and fear in the correlation between social media use and preventive behavior has been testified (indirect effect = 0.04, 95% CI = [0.01, 0.08]).

**Conclusions:**

Social media use might influence the adoption of preventive behaviors through triggering situation awareness and fear. Therefore, health communication regarding COVID-19 prevention should target people with less internet access and low eHealth literacy. Understanding the positive role of negative crisis emotions during outbreaks could also help communicators and policymakers develop appropriate strategies to make people proactive to avoid the remaining health hazard.

## Introduction

Recent years have witnessed a series of epidemics such as Ebola, Zika and H1N1 around the globe, which have also shed substantial light upon the importance of effective health information management (1). In recent epidemics and outbreaks, people's reliance on online media and social networking sites are increasingly heavy especially when information from public health organizations was farfetched (2). Extant scholarship has examined that online news offers considerable diversity in content and sources, and can be updated in real time (3). In addition, the internet provides unique and unfiltered information including emotional support based on users' observations and experiences (4). For individuals seeking more detailed information, such alternative information channels like social media will be considered beneficial to enhance health communication (5).

Coronavirus disease 2019 (COVID-19) is a virus which spread rapidly and caused widespread public panic and dealt a heavy blow to social stability (6). In light of the situation that everyone is spending copious amounts of time indoors, social media usage has surged, which may affect people's psychological states (7). In view of the unique situation, we have the opportunity to study the impact of social media on people's situational awareness, emotional status and subsequent behavior during a real time epidemic.

Although scholars are paying more attention to the role of social media in epidemics, how the use of social media affects the public's awareness of the emergency, affective responses and protective behaviors still need to be fully explored. To fill this gap, this study explored how the use of social media is associated with situational awareness and emotional response, thereby predicting protective behavior. Based on data collected during the 2020 outbreak of COVID-19 in China, this article clarified three influencing paths: (1) how social media use correlated with situation awareness, (2) the extent to which social media use and situation awareness affected crisis emotions like anxiety and fear, and (3) how social media use predicted preventive behaviors through cognitive (i.e., situation awareness) and psychological (i.e., crisis emotions) mechanisms.

### Social Media Use and Situation Awareness in Epidemics

When being caught up in the epidemics, first-hand and accurate health information about emergency risks may not be easily available (8–10). With the development of the internet and communication technologies, the channels that people rely on during an epidemic have changed. Previous studies have shown that online news platforms as well as social media channels are now favored during a public health crisis (11). Media dependency theory also suggested that people's reliance on media depends on information need, and individuals' dependence on media can increase when threats arise in the surrounding environment (12). Specifically, some scholars have suggested that Chinese citizens tend to rely more on the internet and short message texting to resolve ambiguity during SARS epidemic, which seems to be similar with the context of current study (13).

Situation awareness refers to the perception of various elements in the environment within a certain time and space, including the understanding of their meaning and the prediction of their state in the near future, which has been examined in public health emergencies (14, 15). Sarter and Woods defined it as all the available knowledge that can be synthesized to help people assess a situation (16). Drawing on the research by Slovic, knowledge and familiarity can be seen as two important dimensions of situation awareness. Knowledge is about people's perception of how well they know about current situation, and familiarity refers to the extent to which individuals have been accustomed to status quo (17). In a dynamic uncertain environment like epidemics, information and knowledge are also related with how people perceive risks and threats (18). Li and Cao suggested that perceived threat is a component of situation awareness in crisis, which can offer “a logical illustration of the dynamic process of how information is gathered and processed” (15).

Studies found that media information is a source of perceived reality (19), and social media provides an optimal environment to share information rapidly and vividly, making information more tangible and interactive (11). People could find multifaceted risk related information and personal experience from other users, which may spur their awareness (20). The instant news updates from online social networks are likely to attain higher situation awareness for people (15). Therefore, the following hypothesis is formulated:

H1: Social Media Use Will Be Positively Associated with Situation Awareness.

### The Role of Situation Awareness in Shaping Anxiety and Fear

Slovic has found that the degree of familiarity with a risk situation is related with public panic (17). In this sense, people's perception of the prevalence and seriousness of a hazard may help them consider how vulnerable they are, and the representation of the hazard in their mind is inextricably related with feelings (21). For example, van der Meer and Jin have proposed that information in a public health emergency can alter how people estimate the severity of the crisis and evoke negative emotional responses like anxiety, fear and confusion (22). And also, situation awareness represented the cognitive processes, which could be seen as key indicators of affective changes. Findings on attitudes, social perception and emotion also demonstrated that the recognition after processing certain information may be related with affection (23).

According to Appraisal Tendency Framework (ATF) proposed by Lerner and Keltner, discrete emotions have different associations with cognitive appraisal, which means that emotions can be evoked by cognitive evaluation and can inspire people to estimate future events consistent with the evaluation (24). It provides a theoretical base for us to investigate whether crisis emotions are related with recognition of the status quo. Scholars have suggested that COVID-19 epidemic in China has aroused various emotional responses and psychological problems like anxiety and fear (25). Anxiety is cognitively closer to the threat of survival and uncertainty while fear is usually linked with a pessimistic view of concrete and sudden threats, both of which have been demonstrated among individuals during public health outbreaks (26). Although research on situation awareness has provided great insight into how it affects public perception of an epidemic, it still needs to fill in some theoretical and empirical gaps, for example, how people shape their crisis emotions based on situation awareness. We thus propose following hypotheses:

H2-1: Situation Awareness Will Be Positively Associated with Anxiety.
H2-2: Situation Awareness Will Be Positively Associated with Fear.

### Effects of Social Media Use on Crisis Emotions

An infectious disease outbreak can trigger public expression of concerns and elicit negative emotions, especially through social media usage (9). The social media crisis communication model also showed that exposing to crisis information through social media can influence public anticipation of organizational responses and what crisis emotions people are likely to feel (27). Considering that individuals are increasingly use social media during outbreaks and it is important to understand how people consume information via social media, we proposed that anxiety and fear can be elicited by the recognition of crisis situation as a result of social media exposure:

H3-1: Social Media Use Will Have an Indirect Effect on Anxiety through Situation Awareness.
H3-2: Social Media Use Will Have an Indirect Effect on Fear through Situation Awareness.

### Situation Awareness and Crisis Emotions as Antecedents to Protective Behaviors

Protective behavior should be viewed as any behavior performed by an individual to protect, promote and maintain the health status and decrease the probability of getting illness (28). Situation awareness is associated with the evaluation of the actuality and severity of an outbreak of an epidemic, which can be considered as cognitive responses to the pandemic (29). Protective motivation theory suggested that people may take some protective behaviors to avoid negative health consequences based on the assessment of the situation, the severity of potential harm, and the effectiveness of actions (30). Therefore, situation awareness can be the first and foremost factor to encourage protective behaviors during epidemics and outbreaks. The reason why situation awareness can predict protective behavior is because it drives people's needs to manage threats and risks (31). Li and Cao also found that protective behaviors are positively predicted by situation awareness in terms of perceived knowledge and threat (15). In epidemics and outbreaks, people need to collect useful information to make decisions on how to protect themselves (32, 33), so it is necessary to carefully investigate the effect of situation awareness and to understand the consequences of information processing. We thus formulate following hypothesis:

H4: Situation Awareness Will Be Positively and Directly Associated with Protective Behaviors.

Apart from cognitive dimension mentioned above, the emotional responses of epidemics, which refer to the degree of worry, distress or dread that a person feels, are also important factors during crises (29, 34). For example, affective components like feelings of threat, concern and worry are found to be able to directly drive protective behavior in H1N1 influenza (35). The associations between depressive symptoms like anxiety and fear and healthcare-seeking behavior have been studied in the medical domain (36). Specifically, psychologists found that there is a positive association between anxiety and health behavior which opens up a step for us to test the effect of anxiety on protective behavior (37). In terms of fear, previous research demonstrated when the levels of fear are too high, it may hinder engagement with protective behavior (30). However, fear appeals can be powerful persuasive devices if they induce strong perceptions of threat and efficacy with regard to health protective behavior (38). Therefore, we assume that precautionary behaviors can be triggered by anxiety and fear:

H5-1: Anxiety Will Be Positively Associated with Protective Behaviors.
H5-2: Fear Will Be Positively Associated with Protective Behaviors.

Existing studies have found that social media platforms have crucial influence on public perceptions of an infectious disease, which in turn are important elements in predicting individuals' health preventive behaviors (39). While extant scholarship has shed light on how people promote health behaviors because of media exposure, only a few studies have examined both in cognitive and affective ways (40). Therefore, we tried to explore how situation awareness elicited by social media use affected crisis emotions and protective behavior during COVID-19. Therefore, we hypothesized:

H6-1: Social Media Use Will Have an Indirect Effect on Protective Behaviors through Situation Awareness and Anxiety in Serial.
H6-2: Social Media Use Will Have an Indirect Effect on Protective Behaviors through Situation Awareness and Fear in Serial.

**Figure 1:**
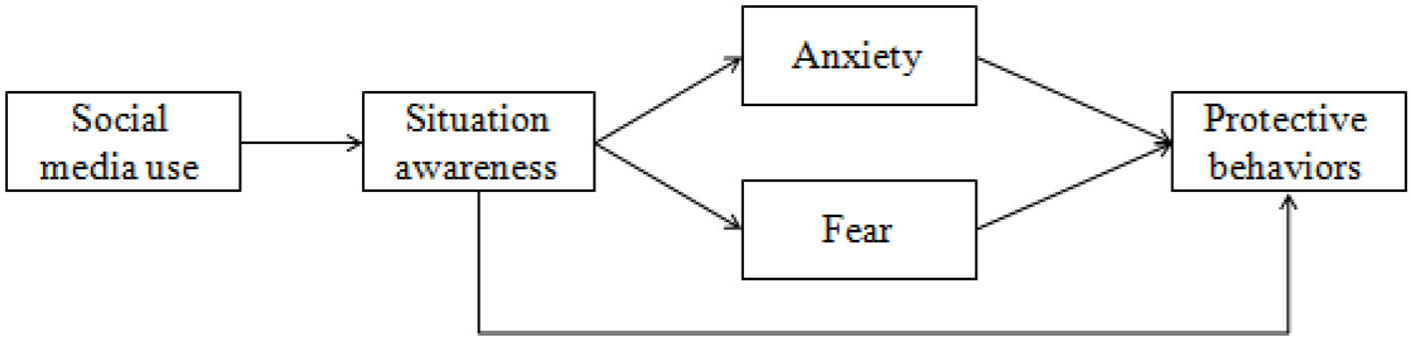
Hypothesized model.

The hypothesized model is shown in Figure 1.

## Method

### Research Design and Recruitment of Participants

This study conducted a cross-sectional survey via an online survey platform “Wenjuanxing” (http://www.sojump.com) to collect data targeting on the mainland Chinese residences over 18 years old. Using the sample service provided by the platform, respondents were randomly invited from a sample dataset up to 2.9 billion people. A total of 632 valid questionnaires were eventually recovered after the check of quality, and the rate of valid recovery was 89.14%. Quotas were set for age (9% 18–24, 19% 25–34, 18% 35–44, 24% 45–54, 22% 55–64, 8% 65+), and there were 376 female and 256 male participants. All the participants are provided with the informed consent form, with an explanation on the research process, potential risks and benefits, principles of anonymity and confidentiality and the right to withdraw. This research was conducted between February 1 and March 1, 2020, when the COVID-19 epidemic caused global attention and China had already begun to issue alarms.

### Measures

#### Exposure to COVID-19 Information Through Social Media (Hereafter, Social Media Use)

We used a 5-point Likert scale (1 = not at all, to 5 = to a large extent) to assess participants' exposure to COVID-19 related information through social media in the following question: “How often have you seen information about COVID-19 on social media?”

#### Situation Awareness

Adapted from researches conducted by previous studies (15, 41), situation awareness was measured by six items using 5-point Likert scale (1 = strongly disagree, 5 = strongly agree): “I understand the common symptoms of COVID-19 include fever, dry cough, fatigue, loss of appetite, loss of smell, and body ache”; “I understand transmission of the COVID-19 can occur by direct contact with infected people and indirect contact with surfaces in the immediate environment or with objects used on the infected person”; “I understand the preventive actions against COVID-19 are basically to wash hands often, avoid close contact, cover coughs and sneezes, clean and disinfect frequently touched surfaces, and monitor my health daily”; “It is possible that I will be affected by COVID-19”; “I believe that the COVID-19 epidemic is severe”; “I think the COVID-19 epidemic threat is immediate” (Cronbach's α = 0.76).

#### Anxiety

We used two items to assess respondents' levels of anxiety ranging from 1 = not at all to 5 = to a large extent (42). The statements included “I feel anxious in the face of COVID-19” and “COVID-19 makes me feel worried” (Cronbach's α = 0.72).

#### Fear

To measure fear about COVID-19, we used the following two items on a 5-point Likert scale ranging from 1 = not at all to 5 = to a large extent (30). The statements were “I am frightened because of COVID-19” and “I feel fearful about COVID-19” (Cronbach's α = 0.71).

#### Protective Behaviors

Taking the research conducted by Lee-Baggley and colleagues as reference, we measured protective behaviors with 11 questions on a 5-point Likert scale (1 = never do, 5 = always do) (42). Instructions to respondents for the protective behavior were “To avoid getting COVID-19, I have personally…” and the example items were “avoided gatherings of people”, “wearing a mask”, and “washing hands more often”. We averaged all the items to get an index of protective behaviors (Cronbach's α = 0.86).

### Analysis

To test the hypotheses in the proposed model, we used SPSS 22.0 to conduct a series of descriptive, bivariate correlation and regression analyses (see Table 1). For control purposes, we linked demographic variables such as gender, age, education, living areas (urban or rural) and income to all the endogenous variables. Mediation analyses were conducted with the help of model 4 and model 6 in PROCESS macro. We used 5,000 bootstrap sample to calculate the indirect effects and confidence intervals.

**Table 1:** Descriptive statistics, inter-correlations and internal consistency of key variables.

	**Mean (SD)**	**Cronbach's α**	**1**	**2**	**3**	**4**	**5**
1. Social media use	4.41 (0.77)	_	_				
2. Situation awareness	4.03 (0.56)	0.76	0.74^**^	_			
3. Anxiety	4.17 (0.64)	0.71	0.73^**^	0.83^**^	_		
4. Fear	3.74 (0.64)	0.72	0.69^**^	0.77^**^	0.91^**^	_	
5. Preventive behavior	4.37 (0.54)	0.86	0.71^**^	0.72^**^	0.66^**^	0.66^**^	_

** *Correlation is significant at the 0.01 level*.

## Results

**Figure 2:**
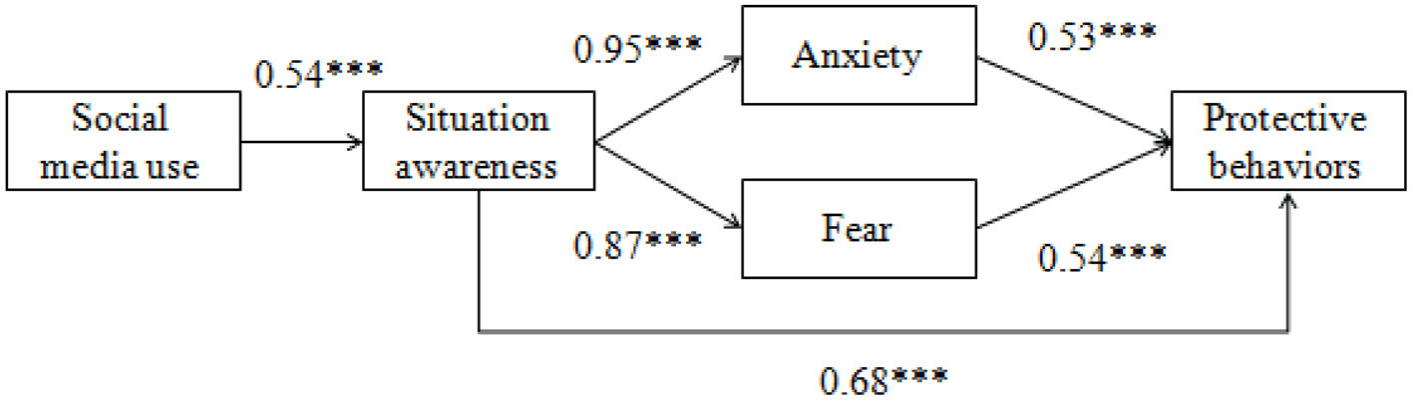
Results of hypothesized model. *** *p* < 0.001.

To test H1-H5, regression analyses were run and the results were shown in Figure 2. As seen from Figure 2, H1 tested whether social media use would be positively associated with situation awareness. The results showed that H1 was supported (B = 0.54, *p* < 0.001).

H2 explored whether situation awareness will affect two self-relevant emotions including anxiety (H2-1) and fear (H2-2). The results demonstrated that both of anxiety (B = 0.95, *p* < 0.001) and fear (B = 0.87, *p* < 0.001) were positively associated with situation awareness. Thus, the results supported H2.

H3 predicted two significant indirect links between social media use and two emotions—anxiety and fear via situation awareness. Specifically, H3-1 supposed that social media use would have an indirect effect on anxiety through situation awareness. The results indicated that the indirect effect was significant (indirect effect = 0.40; 95% CI = [0.34, 0.46]). Therefore, H3-1 was verified. H3-2 examined the indirect effect of social media use on fear through situation awareness. The significance of the mediating role of situation awareness also manifest (indirect effect = 0.35; 95% CI = [0.29, 0.42]), which supported H3-2.

H4 postulated that situation awareness would have a significant effect on preventive behavior, and the effect has been proved to exist (B = 0.68, *p* < 0.001), so H4 was supported.

Similarly, H5 predicted that anxiety (H5-1) and fear (H5-2) would be positively related to preventive behavior. The results indicated that both anxiety (B = 0.53, *p* < 0.001) and fear (B = 0.54, *p* < 0.001) were positively associated with preventive behavior, which supported H5.

H6 explored two indirect paths about the effects of social media use on preventive behavior through situation awareness and two kinds of emotions. Table 2 summarized the indirect effects of the hypothesized mediators. Specifically speaking, H6-1 asked about whether situation awareness and anxiety sequentially mediate the path between social media use and preventive behavior. The results demonstrated that only situation awareness served as a mediator (indirect effect = 0.21, 95% CI = 0.14, 0.27), while situation awareness and anxiety did not mediate the relationship between social media and preventive behavior serially (indirect effect = 0.02, 95% CI = [-0.02, 0.06]), so H6-1 was not supported. H6-2 investigated the serial mediation effect of situation awareness and fear in the correlation between social media use and preventive behavior. As shown in Table 2, the results verified the serial mediation effect (indirect effect = 0.04, 95% CI = [0.01, 0.08]), so H6-2 was supported.

**Table 2:** Results of serial mediation analyses.

		**Point estimation (SE)**	**CI**
**Mediators: situation awareness, anxiety and fear**	**Indirect effect**		
	Via M1, M2	0.02 (0.02)	[−0.02, 0.06]
	Via M1, M3	0.04 (0.02)^a^	[0.01, 0.08]

a *indicates significant effects*.

*^b^M1 = Situation awareness, M2 = Anxiety, M3 = Fear. ^c^CIs are bias-corrected 95% confidence based on 5,000 bootstrap samples*.

## Discussion

### Principal Results

As social media has become important sources of information during outbreaks of infectious diseases, some health communication literature attempts to clarify the dynamic relationship between social media use and preventive behaviors (40). During epidemics and outbreaks, the use of social media will not only be connected with our understanding of infectious diseases, but also have the potential to cause our emotional changes, which are crucial in shaping public self-protective behavior (26, 40). Following those lines of enquiry, this study aimed to advance current knowledge surrounding the relationship between social media use, people's cognition, affection and health protective behaviors during epidemic outbreaks. Specifically, it did so in three ways: first, by exploring how social media exposure to COVID-19 risks related information was correlated with situation awareness; second, by exploring how social media risk information exposure and situation awareness could be related with crisis emotions; third, how social media use could be correlated with protective behaviors through situation awareness and negative emotional responses. The correlation study of current research mainly drew two possible results—first, social media use could affect preventive behavior through situation awareness and the negative crisis emotion; second, people who were prone to take preventive behavior with higher level of situation awareness and crisis emotion would be more positive in social media use.

Taking the first scenario into consideration, our results suggest that social media use can help gathering and dispersing relevant and useful information, which help the public be more aware of the situation they are faced with. Guided by previous scholarship on situation awareness (42–44), the current study suggests that exposure to crisis information through social media is positively related with situation awareness which also predict preventive behavior for the public. Previous studies have shown that general risk information has a limited impact on protective behavior, while personalized risk feedback is more likely to trigger preventive intentions and behaviors (45). Extending previous findings, this study pointed out the positive side of negative crisis emotions during outbreaks, making people be proactive to avoid the remaining health hazard. Moreover, this study suggested that social media use have the potential to predict individuals' preventive behavior through the serial multiple mediation role of situation awareness and fear instead of anxiety. This finding demonstrates that different emotions appeared to play different mediation roles, which corresponds to extant scholarship indicating that how people respond to the risk might be subject to the effects of emotional valence (28). It might be speculated that even if social media use may trigger numerous crisis emotions, fear might play a stronger role than other kinds of emotions in shaping people's cognition or behavior (46).

Secondly, the results also hinted that people who are more willing to take preventive actions and who are more emotionally sensitive during COVID-19, are also more active in social media use. Indeed, social media has been widely accessed among the population around the whole world (47), and the social distancing condition during COVID-19 pandemic even changed the original social interaction pattern (48). As we have found that people's crisis emotions are closely related with social media use, future research are encouraged to explore whether social media can cause negative emotions or people are using it for social inclusion and connection to get rid of negative emotions. In this sense, social media may have the potential for offering relief during epidemics (49). In addition, the finding that active self-protectors also used social media more willingly not only highlighted the key role of social media in the context of fighting against health pandemic, but also reminded us to pay more attention to people with less access to internet as well as social media. Health communication strategies should not only be implemented on social media channels, but also be executed on other scenarios including mass media platforms and interpersonal communications (e.g., doctors, families, etc.) (50).

### Comparison With Prior Work

The current study highlighted the potential roles of social media in shaping people's cognitions, emotions and behaviors, which corresponds to previous research indicating that crisis information exposure through social media channels can trigger public expression of concerns and elicit negative emotions (40). This study also suggests that as a platform for information exchange and a space for contagious sentiments, social media highly attract people who are more willing to take protective measures and more sensitive during epidemics. Therefore, future studies are encouraged to explore the causality between using social media and emotional as well as behavioral responses. In addition, it should be noted that previous studies argue that mass media can also make cognition and emotion responses during risks more salient than usual (51), which leave a possibility of comparison for future research to consider. When framing the crisis as a disaster, a general health issue organization relied more on traditional media than social media (11), so it would be more valuable to test the effect of other media channels as well.

### Limitations

Single-item measures have had bad reputation, while a series of recent published articles have done much to alleviate the stigma surrounding single-item measures (52). Arguments against single-item measures include assertions that single-item measures have uncertain reliability and lack the capacity to conduct finer-grained assessment (52). Therefore, developing multi-item measurements for social media use is warranted for bettering current research. Also, studies focusing on various activities through social media channels could help interpret specific influence of social media during epidemic. Nevertheless, some scholars argue that the use of single-item measure should not necessarily be considered unreliable when the construct is unambiguous and narrow in scope (53, 54). Furthermore, using single-item measures could save administration time, avoid confusion and improve the recovery rate of questionnaires (52), and it is interesting to note that single-item measures are not less valid or reliable than their multi-item counterparts in health and psychological research domains (55, 56). In our research and survey, social media use is a general behavior and a unidimensional, clearly defined and unquestioned construct, which made it not a serious threat to the confidence of our results. Secondly, the cross-sectional data we used does not allow testing for causality. However, we used a large enough dataset to minimize the likelihood of Type One error and ensure that findings are valid and reliable. According to Spector, the cross-sectional design has its merits and can indicate the relationships among research variables (57). To improve the current methodology, in-depth interviews involved with more interpretation on the effects of social media use can be taken into consideration. We also think current findings can be a valuable reference for future studies interested in a longitudinal study design. Although our research is not flawless, we believe our findings can provide essential perspectives as a starting point and can inform how subsequent research can be conducted in a more robust way. It is also suggested that further research is needed to determine the causality. Thirdly, our research mainly focused on the crisis emotions including anxiety and fear. For future research, it will be worthwhile to investigate other types of crisis emotions such as anger, which has been proved to be important factors in public risk perception during pandemics (40).

## Conclusions

The findings of current study highlighted the distinct roles of social media in the outbreak of an epidemic from a cognitive and emotional perspective, thereby adding new knowledge to health communication strategies and cognitive psychology paradigm. Such an attempt to integrate relevant theories and factual evidence in a coherent way can advance the development of risk management in a public health emergence. Future research is needed to examine why social media have the potential to shape and affect public perception of an epidemic, for example through personal narratives and vivid visual imagery timely, and to identify precise mechanisms based on which it does so. Hopefully the current study can help health communication practitioners better understand the process of how public cognitive, emotional and behavioral responses are related with social media and develop effective risk communication strategies.

## Data Availability Statement

The raw data supporting the conclusions of this article will be made available by the authors, without undue reservation.

## Ethics Statement

The studies involving human participants were reviewed and approved by Shanghai Jiao Tong University. The patients/participants provided their written informed consent to participate in this study.

## Author Contributions

All authors listed have made a substantial, direct, and intellectual contribution to the work and approved it for publication. All authors contributed equally.

## Funding

This project is sponsored by National Social Science Foundation of China (18ZDA312) and National Key R&D Program of China (2020YFF0305300). We are also grateful to the support provided by the Palace Museum Open Project (2021), and we appreciate the helpful comments and suggestions from anonymous reviewers and journal editors.

## Conflict of Interest

The authors declare that the research was conducted in the absence of any commercial or financial relationships that could be construed as a potential conflict of interest.

## Publisher's Note

All claims expressed in this article are solely those of the authors and do not necessarily represent those of their affiliated organizations, or those of the publisher, the editors and the reviewers. Any product that may be evaluated in this article, or claim that may be made by its manufacturer, is not guaranteed or endorsed by the publisher.
