# Supplementary figures and images for: Exploring the Mediating Role of Situation Awareness and Crisis Emotions Between Social Media Use and COVID-19 Protective Behaviors: Cross-Sectional Study
Source: Front Public Health. 2022 Apr 28;10:793033. doi: 10.3389/fpubh.2022.793033 (PMC9096136; doi:10.3389/fpubh.2022.793033)

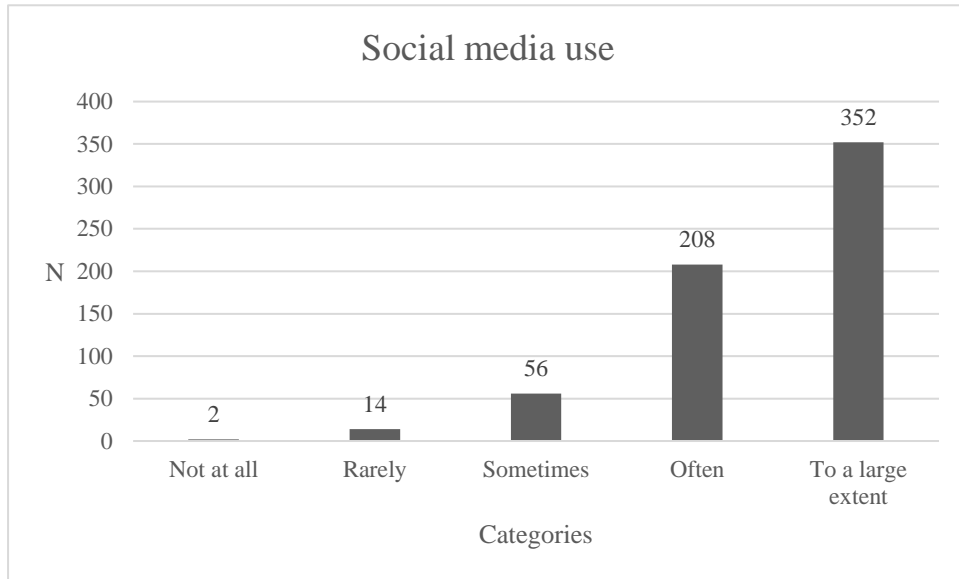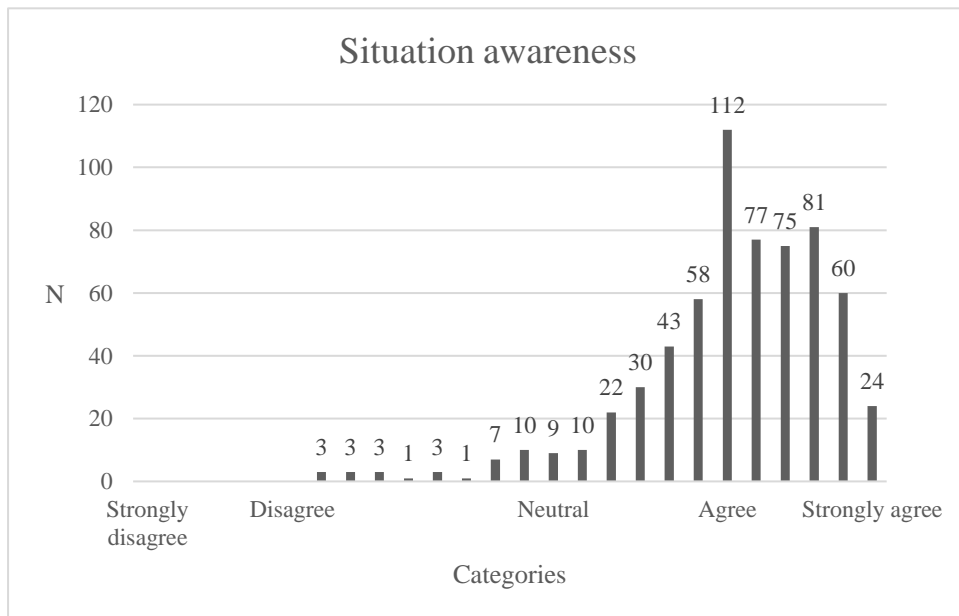

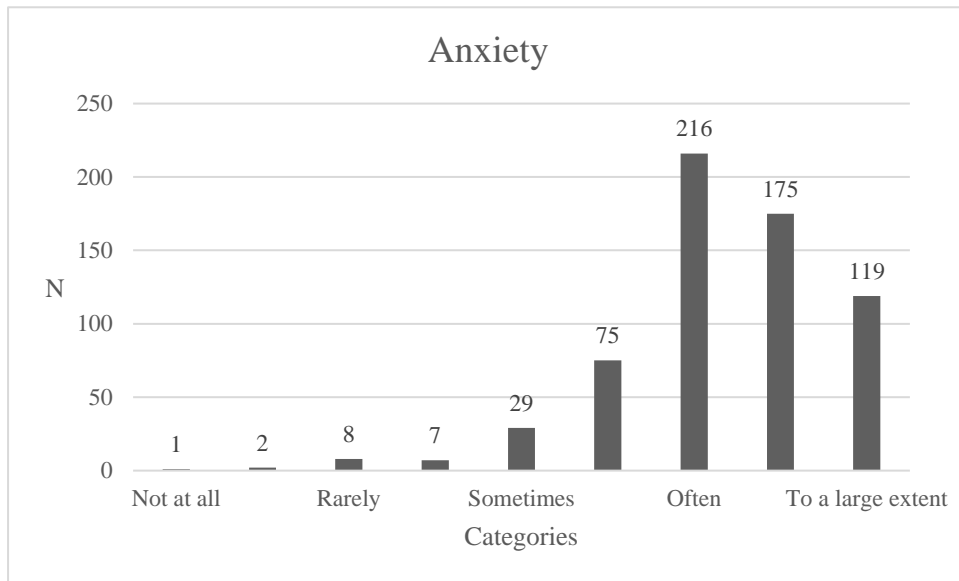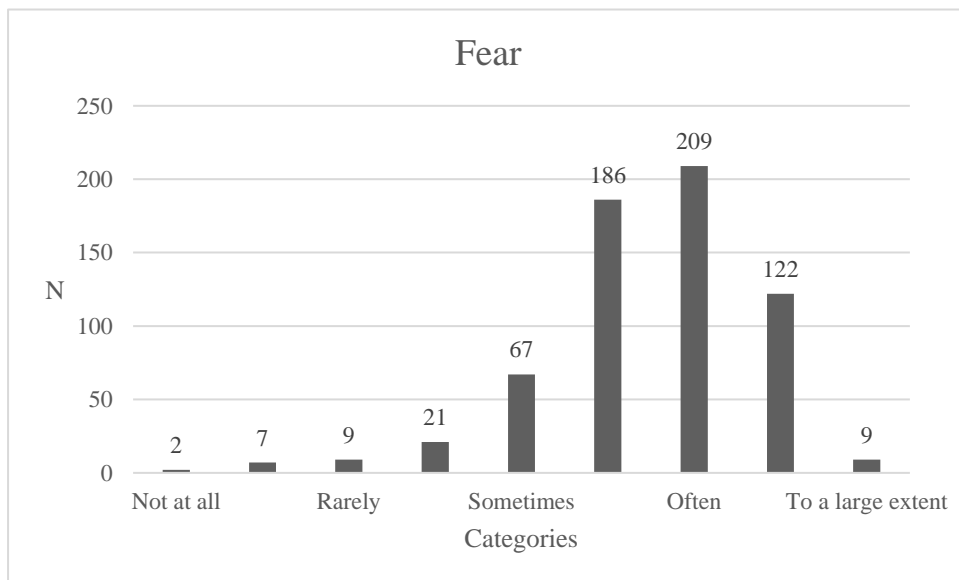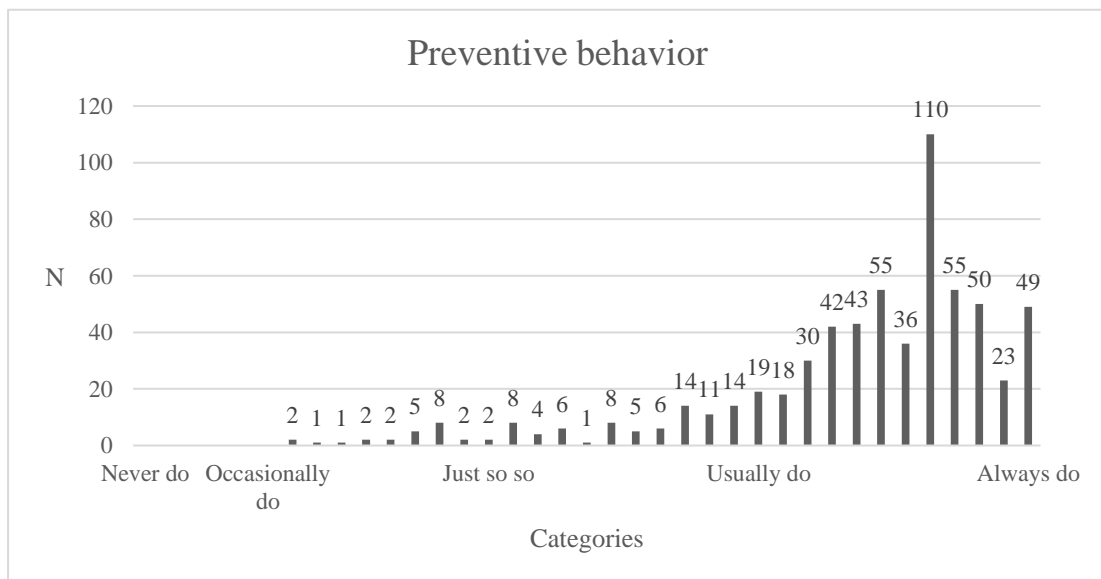

Supplement: Supplementary file 1 [file Data_Sheet_1.pdf]
