# Supplementary material for: Exploring the Mediating Role of Situation Awareness and Crisis Emotions Between Social Media Use and COVID-19 Protective Behaviors: Cross-Sectional Study
Source: Front Public Health. 2022 Apr 28;10:793033. doi: 10.3389/fpubh.2022.793033 (PMC9096136; doi:10.3389/fpubh.2022.793033)
Supplement: Supplementary file 2 [file Data_Sheet_2.pdf]

A Survey on Social Media Use and COVID-19 Prevention

1. In the past week, how often have you seen information about COVID-19 on social media?

1) Not at all
2) Rarely
3) Sometimes
4) Often
5) To a large extent

2. **Read the following sentences and choose the number that most fits your idea. The bigger the number is, the more you agree with it** *(1 = strongly disagree, 5 = strongly agree).*

	strongly disagree → strongly agree				
1) I understand the common symptoms of COVID-19 include fever, dry cough, fatigue, loss of appetite, loss of smell, and body ache.	1	2	3	4	5
2) I understand transmission of the COVID-19 can occur by direct contact with infected people and indirect contact with surfaces in the immediate environment or with objects used on the infected person.	1	2	3	4	5
3) I understand the preventive actions against COVID-19 are basically to wash hands often, avoid close contact, cover coughs and sneezes, clean and disinfect frequently touched surfaces, and monitor my health daily.	1	2	3	4	5
4) It is possible that I will be affected by COVID-19.	1	2	3	4	5
5) I believe that the COVID-19 epidemic is severe.	1	2	3	4	5
6) I think the COVID-19 epidemic threat is immediate.	1	2	3	4	5

3. Read the following sentences and choose the number that most fits your idea. The bigger the number is, the more you agree with it *(1 = not at all, 5 = to a large extent).*

	not at all → to a large extent				
1) I feel anxious in the face of COVID-19.	1	2	3	4	5
2) COVID-19 makes me feel worried.	1	2	3	4	5

4. Read the following sentences and choose the number that most fits your idea. The bigger the number is, the more you agree with it *(1 = not at all, 5 = to a large extent)***.**

	not at all → to a large extent				
1) I am frightened because of COVID-19.	1	2	3	4	5
2) I feel fearful about COVID-19.	1	2	3	4	5

5. **How often did you take following measures for preventing COVID-19? Please choose the number that most applies to your situation. The bigger the number is, the more often you do it.** *(1 = never do, 2 = occasionally do, 3 = just so so, 4 = usually do, 5 = always do).*

To avoid getting COVID-19, I have personally…	never do → always do				
1）avoided travel to COVID-19 infected areas.	1	2	3	4	5
2) avoided travel on subways or commuter trains.	1	2	3	4	5
3) avoided eating in food courts/food centers.	1	2	3	4	5
4) not gone to work/school.	1	2	3	4	5
5) avoided a person who has a fever.	1	2	3	4	5
6) avoided a person you know has just come from an area infected with COVID-19.	1	2	3	4	5
7) avoided a person who has a family member who has come down with COVID-19.	1	2	3	4	5
8) used disinfectants.	1	2	3	4	5
9) washed hands more often.	1	2	3	4	5
10) wearing a mask.	1	2	3	4	5
11) taken an herbal supplement.	1	2	3	4	5

6. **What's your gender?** 1）male 2）female

7. **When were you born? (Fill in the blanks by yourself, such as 1980)**

8. **What is your final educational level?**

1) Middle school or less
2) High school or secondary vocational school
3) Associate degree
4) Bachelor degree
5) Master and above

9. How about your monthly income generally?

1）Under￥1,500
2）￥1,500 - 3,000
3）￥3,001 - 5,000
4）￥5,001 – 8,000

5）￥8,001 - 12,000
6）￥12,001 – 20,000
7) ￥20,000 above

10. What is your marriage / residence status?

1) Single
2) Divorced or widowed
3) Separated
4) Cohabiting
5) Married

Thanks for your answers and support!
